# Satisfaction of psychologically impaired patients with health-care services: A Saudi Arabian perspective

**DOI:** 10.3389/fpubh.2022.1000833

**Published:** 2022-09-29

**Authors:** Majid Alhomrani, Walaa F. Alsanie, Osama Abdulaziz, Magdi M. Salih, Abdulwahab Alamri, Syed Mohammed Basheeruddin Asdaq, Abdulhakeem S. Alamri

**Affiliations:** ^1^Department of Clinical Laboratory Sciences, The Faculty of Applied Medical Sciences, Taif University, Taif, Saudi Arabia; ^2^Centre of Biomedical Sciences Research (CBSR), Deanship of Scientific Research, Taif University, Taif, Saudi Arabia; ^3^Department of Pharmacology and Toxicology, College of Pharmacy, University of Hail, Hail, Saudi Arabia; ^4^Department of Pharmacy Practice, College of Pharmacy, AlMaarefa University, Riyadh, Saudi Arabia

**Keywords:** patients' satisfaction, PSQ-18, pharmacy services, hospital services, psychological ailment

## Abstract

Psychological problems affect a sizable portion of the population, and they require special care. In the current study, we aimed to assess patient satisfaction with the healthcare system at one of the multispecialty hospitals in Riyadh, Saudi Arabia, as well as to identify potential factors that can have an impact on patient satisfaction. A validated pre-tested questionnaire including features to evaluate general hospital services (HS-6 items), nursing services (NS-3 items), pharmacy services (PS-7 items), and a standard patient satisfaction questionnaire (PSQ-18 item) was administered to patients who had been receiving therapy for their psychological disease for the past 3 months. Using binary and multiple regression analysis, the strengths of the associations between sociodemographic factors and patient satisfaction measures were evaluated. The results were expressed as adjusted odds ratios (AOR), which were deemed significant when the *P* value was < 0.05. Sixty-six percent of the 258 study participants were men, and sixty percent of them were between the ages of 18 and 35 years. The bulk of survey respondents (74%) were employed, married, and well–educated. Our research revealed that those who were employed (AOR, HS-2.5; NS-2.65, PS-2.32), have a higher education (AOR, HS-2.23, NS-2.63, PS-2.82), male gender (AOR, HS-1.12, NS-1.08, PS-1.86) and between the ages of 18 and 35 years (AOR, HS-1.48, NS-1.53, PS-1.67) were more likely to be satisfied with general hospital, nursing, and pharmacy services. Further, those who were married had 1.43 and 1.21 times more chance of satisfaction with the pharmacy and nursing services, respectively, compared to singles. Additionally, those with employment had odds of being satisfied that were 2.4 times higher, highly educated individuals had odds that were 2.1 times higher, participants between the ages of 18 and 35 had odds that were 1.51 times higher, and men had odds that were 1.41 times higher on the patient satisfaction questionnaire scale (PSQ-18). Overall, the study participants' satisfaction with general hospital, nursing, and pharmacy services was 70, 76.3, and 83.3%, respectively, compared to only 61.2% on the PSQ-18. Participants in the survey awarded the hospital amenities, pharmacy services, and nursing care high ratings. The medical care, however, fell short of expectations. The study's findings suggest that action needs to be taken to enhance healthcare system services, particularly in the psychological departments of the medical organization.

## Introduction

Satisfaction of patients refers to the patients' feelings about the services provided by the health care system to satisfy their needs (1). Currently, the health care system has metamorphosed from “healthcare provider-centered” to “patient-centered,” reflecting the idea of giving satisfactory services to patients. Measurement of patient satisfaction is an important area of healthcare research published in several parts of the world (2–4). Patient satisfaction has gradually developed as an outcome measurement for evaluating and improving health and care services (5). It is a special form of consumer attitude—that is, a post-experience phenomenon reflecting how much a patient liked or disliked the service (6). It has been a widely recognized indicator of the quality of care in medical practice (7). Patient satisfaction is also shown to have positive implications for health care institutions, including patient retention and developing a reputation in the community (8). It is reported that achieving better satisfaction has a direct association with the reputation of the healthcare center (9). Overall, patient satisfaction has become an essential key performance indicator across accrediting bodies (10).

With a growing interest in quality assurance and comparisons at the global level, assessing healthcare satisfaction has begun to make its way into many transitional cultures, as well as in countries that are commonly referred to as “third world”, “developing”, or “emerging economies”. Many criteria that are typically reported by patients were regularly measured to determine patient satisfaction, and the results were then examined by researchers to implement new programs and policies that hoped to increase patient satisfaction and lead to improved outcomes (11, 12). These factors include the services offered at the hospital settings, services extended by health care professionals including nurses, pharmacist, and medical doctors, in addition to the infrastructure and general appearance of the hospital (13, 14). However, research investigating psychiatric care satisfaction in the Arabian Gulf, particularly Saudi Arabia, is very scarce. As one of the Arab world's most populous countries, it is critical to promote a high-quality healthcare system. Several studies have indicated the growing level of psychological disorders among people across the globe. Psychiatric disorders are the fourth leading cause of the world's disease burden (15). A study done in Saudi Arabia reported the prevalence rate of psychiatric disorders at a range of between 30 and 46% (16).

It is acknowledged that gauging patient satisfaction with mental healthcare services is a crucial component in evaluating the effectiveness and efficiency of clinical service delivery (17). Reduced treatment compliance results from low patient satisfaction with mental healthcare, which may ultimately have negative health effects (18). One of the biggest challenges for mental health facilities is providing long-term mental healthcare for people with mental diseases that satisfies their expectations. 13 Despite the significant burden of mental diseases, these illnesses have received less attention in lower- and middle-income nations (19).

Numerous research on psychiatry outpatients in low- and middle-income nations revealed variable degrees of patient satisfaction. A study from Tanzania reported (20) for 70% of high levels of satisfaction, while a study from Egypt claimed 31.2% of highly satisfied mental patients (21). A survey of psychiatric patients in Ethiopia revealed that 61.2% of participants were extremely satisfied with the psychiatric care services (22). According to another study conducted in Ethiopia, 50.3% of individuals with mental illness reported being extremely satisfied, 31% reported being satisfied, and 18.7% reported being dissatisfied (23).

Studies have shown that a variety of factors, including age, gender, types of mental illness, waiting times for services, medication availability, treatment information provided, marital status, medical costs, education, confidentiality, occupation, residency, hospital infrastructure, hospital cleanliness, and respect for patient preferences, all have an impact on how satisfied patients are (24). The quality of care will be improved by knowing the extent of patient satisfaction and the factors related to the psychiatric service. Therefore, the purpose of this study was to ascertain psychologically impaired patients' satisfaction with health-care services provided in one of Saudi Arabia's multispecialty hospitals, as well as to investigate the potential factors that influenced them.

## Materials and methods

### Study location, participants and sampling

This is a three-month cross-sectional study conducted in a multispecialty hospital's outpatient psychiatric department in Riyadh, Saudi Arabia from February to April 2022. The study's participants ranged in age from 18 to 60 years old and were being treated for any psychiatric condition as defined by the Diagnostic and Statistical Manual for Mental Disorders. All the participants were able to comprehend and complete a questionnaire. They were in good enough health, cognitively and emotionally, to give their consent to participate in the study. Participants who were clinically assessed to be “lower functioning,” unable to give permission, and whose cognitive performance indicated moderate to severe cognitive impairment were excluded from the study (25). Training was given to data collectors on the content of the data collection tools and interviewing techniques. The data collection process was supervised and monitored by the research team of this project. Participants were made aware of the study's purpose, methodology, and potential risks, as well as the voluntary nature of their participation and the confidentiality of their responses by the data collector. They were informed that they might refuse or withdraw at any time without suffering consequences. To make it easier for participants to understand and comprehend, all communications with them were conducted in the local Arabic language. Those who agreed to complete the questionnaire were included in the study by means of purposive sampling. The questionnaire was self-administered by the participants. However, a data collector was available at the site to clear up any ambiguity. The study protocol was approved by the research committee of the College of Pharmacy, AlMaarefa University, Riyadh, Saudi Arabia. Using OpenEpi software, the required sample size was calculated after the level of significance was set at 0.05, power at 0.8, and precision at 95 percent confidence interval. The sample size was estimated to be 243.

### Study materials

There were four parts to this questionnaire-based investigation. Section I included items relating to the participants' sociodemographic characteristics, such as gender, age, educational level, employment position, and marital status. The second portion addressed general service satisfaction, which was separated into three sub-sections: (a) satisfaction with registration and reception (two items on registration time and public relations services), (b) general hospital facilities (four items about the hospital's peacefulness, cleanliness, physical facilities, and overall patient opinion of the facility), and (c) nurse care (three items on courtesy, cooperation, and respect). The elements of second portion of the questionnaire were adapted from another published article (26). The third portion included a total of seven items to evaluate patients' satisfaction with the pharmacy services, including medication supply and availability; patient counseling; resolving medication-related problems; setting-specific structural issues; and general pharmacy services. The elements of this section were adapted from validated questionnaire that was previously published (27) after suitable modification. Each variable was rated on a five-point scale and scored (strongly agree-5 score, agree-4 score, neutral-3 score, disagree-2 score, and strongly disagree-1 score).

The final component included a brief form of the typical patient satisfaction questionnaire (PSQ-18) (14). It contains seven different evaluation categories that encompass different aspects of satisfaction. Items 3 and 17 judge general satisfaction, whereas items 2, 4, 6, and 14 gauge the technical quality of services. Items 10 and 11 assessed interpersonal behavior, whereas items 1 and 3 determined communications. Item numbers 5 and 7 screen the financial aspect. Items 12 and 15 measured time spent with the doctor, while items 8, 9, 16, and 18 assessed accessibility and convenience. The responses were tallied on a Likert scale that ranged from strong agreement to strong disagreement, with a high score of 5 indicating a high level of satisfaction with the healthcare services.

The questionnaire was backwards-forward translated into Arabic. The face and content validity of each questionnaire used in the study were checked with the help of team of experts that included psychiatrist, a psychologist, a pharmacologist, and a language expert. Internal consistency tests and item-scale correlations were also conducted to confirm the Arabic version's reliability (28). A pilot study was conducted to see if any items were misunderstood. The Arabic questionnaire's internal consistency was assessed, and Cronbach's reliability was confirmed for seven categories, with Cronbach's coefficients ranging from 0.68 to 0.88.

### Statistical analysis

The sociodemographic characteristics of the patients were enumerated using descriptive statistics. The presentation of all sociodemographic data uses percentages and frequencies. The satisfaction score was calculated based on the ratings accorded by the participants for each item in the different outcome variables. The mean of the scores was determined on a scale of 1–5 (Likert scale). The satisfaction scores for general hospital, nursing, and pharmacy services were statistically compared between male and female participants using Pearson's chi-square. If the *P*-value was < 0.05, it was deemed significant.

The satisfaction score was then divided into two categories: dissatisfaction (below the mean) and satisfaction (equal or above the mean). Binary logistic regression analyses were conducted to ascertain relationships between the various independent factors and the outcome variables (dependent variables). The relationship between independent variables (socio-demographic characteristics such as age, gender, education level, marital status, and employment status) and the degree of patient satisfaction with general hospital, nursing, pharmacy, and patients' satisfaction score in the PSQ-18 score (dependent/outcome variables) was assessed using the adjusted odds ratio. In all the analyses, significance testing was done using two-sided *P* values (P) and 95% confidence levels. In the final model, *P* value < 0.05 was considered statistically significant.

## Results

### Sociodemographic characteristics of the participants

Out of 258 participants recruited in the study, 66% were male and 60% of them were in an age group of 18–35 years (Table 1). Most of the surveyors were highly educated (74%), married (51%) and employed (54%).

**Table 1:** Sociodemographic properties of the participants.

**Variables**	**Number (258)**	**Percentage**
**Gender**
Male	170	66%
Female	88	34%
**Age**
18–35	156	60%
36–50	75	29%
>50	27	10%
**Educational level**
Higher	190	74%
Secondary	68	26%
**Marital status**
Married	132	51%
Single	118	46%
Separated	8	3%
**Employment status**
Employed	139	54%
Student	71	28%
Non-employed	48	19%

### Patient satisfaction with the general hospital and nursing services

When compared to their female counterparts, male patients exhibited a higher degree of satisfaction with most of the healthcare services supplied at the hospital's reception and registration sections, as seen in Table 2 (average, 4.07 Vs. 3.73). Male participants gave the greatest scores to the tranquility of the hospital surroundings, while female participants gave higher ratings to the privacy arrangements. The components used to assess the satisfaction of nursing services received higher ratings than those used to assess the satisfaction of general healthcare services. The ability of nurses to graciously listen to patients received the best scores from the study samples. Although the satisfaction scores given by male participants were higher than females, none of them was statistically significant.

**Table 2:** Satisfaction with the general healthcare and nursing services.

**Questions**	**Male**	**Female**	*P* value*
The registration time in the hospital is reasonable	4.1	3.81	0.092
The public relation services offered in the hospital are satisfactory	3.98	3.78	0.765
The rooms were kept clean	4.18	3.76	0.077
The area is quiet at night	4.23	3.73	0.065
Staff make sure you have enough privacy	4.15	3.85	0.081
It is easy to find your way around the hospital	3.79	3.47	0.231
Nurses treat you with courtesy and respect	4.20	3.92	0.091
Nurses listen to you carefully	4.22	3.81	0.092
Nurses explain things in a way you could understand	4.18	3.82	0.089

*Pearson's chi-square, and Fisher's exact test.

For elements pertaining to general hospital services, the mean satisfaction score was 3.90. The satisfaction score was therefore divided into two categories: satisfaction (above or equal the mean of 3.90) and dissatisfaction (below the mean of 3.90). Table 3 shows that individuals who were employed (AOR, 2.5) reported higher levels of satisfaction with general healthcare services than participants who are unemployed. In addition, subjects with greater education had better odds (AOR, 2.23) of being satisfied than those with lower education. Additionally, men were found to be 1.12 times more satisfied with general healthcare services than women, and those between the ages of 18 and 35 were found to be 1.48 times more satisfied with general healthcare services than people over the age of 50. Overall, 70% of the participants were satisfied with the general health care services offered at the study sites.

**Table 3:** Logistic regression analysis of factors influencing patients' satisfaction on general healthcare services (*n* = 258).

**Variables**	**Frequency (%)**		**AOR (95% CI)**	***P* value**
	**Satisfied** **[181 (70)]**	**Dissatisfied** **[77 (30)]**		
**Gender**	
Male	122 (67.4)	48 (62.3)	1.12 (0.76, 1.92)	0.77
Female	59 (32.6)	29 (37.7)	1.0	
**Age**	
18–35	118 (65)	38 (49.3)	1.48 (1.13, 2.67)	0.042*
36–50	48 (26.5)	27 (35)	0.81 (0.58, 1.34)	0.88
>50	15 (8.2)	12 (15.6)	1.0	
**Educational level**	
Higher	136 (81.7)	54 (61)	2.23 (1.5, 3.4)	0.03*
Secondary	45 (18.3)	23 (39)	1.0	
**Marital status**	
Married	91 (50.3)	41 (53.2)	0.81 (0.51, 2.1)	0.41
Single	90 (49.7)	36 (46.7)	1.0	
**Employment status**	
Employed	121 (66.8)	18 (23)	2.5 (1.6, 3.4)	0.012*
Student	38 (21)	33 (42.8)	0.86 (0.45, 1.7)	0.38
Non-employed	22 (12.1)	26 (33.7)	1.0	

1, showed reference used. *Showed a P < 0.05 in the multivariate analysis and statistically significant, CI (Confidence interval).

The mean satisfaction rating for nursing-related items was 4.05. To distinguish between satisfaction (above or equal the mean of 4.05) and dissatisfaction (below the mean of 4.05), the satisfaction score was dichotomized. More than 76% of the participants were happy with the nursing services provided by the hospital, as indicated in Table 4. Patients who were employed (AOR, 2.65) and had a higher level of education (AOR, 2.63) had higher odds of being satisfied. In addition, patients between the ages of 18 and 35 years (AOR, 1.53), married people (AOR, 1.21), and men (AOR, 1.08) showed higher rates of satisfaction with nursing services in comparison to participants who were above the age of 50, singles, and females, respectively.

**Table 4:** Logistic regression analysis of factors influencing patients' satisfaction on nursing services (*n* = 258).

**Variables**	**Frequency (%)**		**AOR (95% CI)**	***P* value**
	**Satisfied** **[197 (76.3)]**	**Dissatisfied** **[61(23.64)]**		
**Gender**	
Male	134 (68)	36 (59)	1.08 (0.56, 1.97)	0.045*
Female	63 (32)	25 (41)	1.0	
**Age**	
18–35	131 (66.5)	25 (41)	1.53 (1.00, 2.87)	0.029*
36–50	49 (24.8)	26 (42.6)	0.92 (0.38, 1.52)	0.92
>50	17 (8.6)	10 (16.4)	1.0	
**Educational level**	
Higher	165 (83.8)	25 (41)	2.63 (1.32, 3.67)	0.025*
Secondary	32 (16.3)	36 (59)	1.0	
**Marital status**	
Married	114 (58)	18 (30)	1.21 (0.66, 2.45)	0.045*
Single	83 (42)	43 (70)	1.0	
**Employment status**	
Employed	126 (64)	13 (21.3)	2.65 (1.4, 3.78)	0.022*
Student	42 (21.3)	29 (47.5)	0.66 (0.25, 1.2)	0.76
Non-employed	29 (14.7)	19 (31.1)	1.0	

1, showed reference used. *Showed a P < 0.05 in the multivariate analysis and statistically significant, CI (Confidence interval).

### Patient satisfaction with the pharmacy services

Overall, the study samples showed high levels of satisfaction with pharmacy services, with male surveyors giving higher scores than females (Table 5), however, these differences were not statistically significant. Male surveyors gave the highest grades to the quantity of medication provided, while female surveyors were pleased with the pharmacist's explanation. The hygiene of the pharmacy area was praised by both male and female participants.

**Table 5:** Satisfaction with pharmacy services.

**Questions**	**Male**	**Female**	*P* value*
Pharmacist helped to solve any problem getting the medication	4.28	3.96	0.089
Medication quantity supplied to me was sufficient	4.31	4.02	0.651
The pharmacy area was clean and acceptable	4.31	4.10	0.371
Pharmacist explained the reason for my medication	4.19	4.10	0.859
Pharmacists do counsel for the use of medication and its side effect	4.11	3.87	0.093
Overall, i am satisfied with the pharmacy services in the hospital i visit.	4.44	4.25	0.922

*Pearson's chi-square and Fisher's exact test.

The mean satisfaction score for pharmacy services was 4.16. The satisfaction score was therefore divided into two categories: satisfaction (above or equal the mean of 4.16) and dissatisfaction (below the mean of 4.16). The satisfaction of the study participants with the pharmacy services were subjected for regression analysis and given in Table 6. The odds of being satisfied with the pharmacy services provided at the hospital were generally greater among individuals with higher education (AOR, 2.82), among employed people (AOR, 2.32), men (AOR, 1.86), people in their 18–35 years (AOR, 1.67), and married people (AOR, 1.43).

**Table 6:** Logistic regression analysis of factors influencing patients' satisfaction on pharmacy services (*n* = 258).

**Variables**	**Frequency (%)**		**AOR (95% CI)**	***P* value**
	**Satisfied** **[215 (83.3)]**	**Dissatisfied** **[43 (16.7)]**		
**Gender**	
Male	156 (72.5)	14 (32.5)	1.86 (0.76, 2.75)	0.021*
Female	59 (27.5)	29 (67.4)	1.0	
**Age**	
18–35	141 (65.6)	15 (34.9)	1.67 (1.03, 2.89)	0.019*
36–50	52 (24.1)	23 (53.5)	0.52 (0.28, 1.62)	0.96
>50	22 (10.2)	5 (11.6)	1.0	
**Educational level**	
Higher	178 (82.8)	12 (28)	2.82 (1.22, 3.87)	0.04*
Secondary	37 (17.2)	31 (72)	1.0	
**Marital status**	
Married	122 (56.7)	10 (23.2)	1.43 (0.76, 2.24)	0.05*
Single	93 (43.3)	33 (76.7)	1.0	
**Employment status**	
Employed	130 (60.4)	9 (20.9)	2.32 (1.3, 3.99)	0.011*
Student	51 (23.7)	20 (46.5)	0.73 (0.51, 1.31)	0.62
Non-employed	34 (15.9)	14 (32.5)	1.0	

1, showed reference used. *Showed a P < 0.05 in the multivariate analysis and statistically significant, CI (Confidence interval).

### Comparison of patients' satisfaction of pharmacy services with other healthcare services

**Figure 1:**
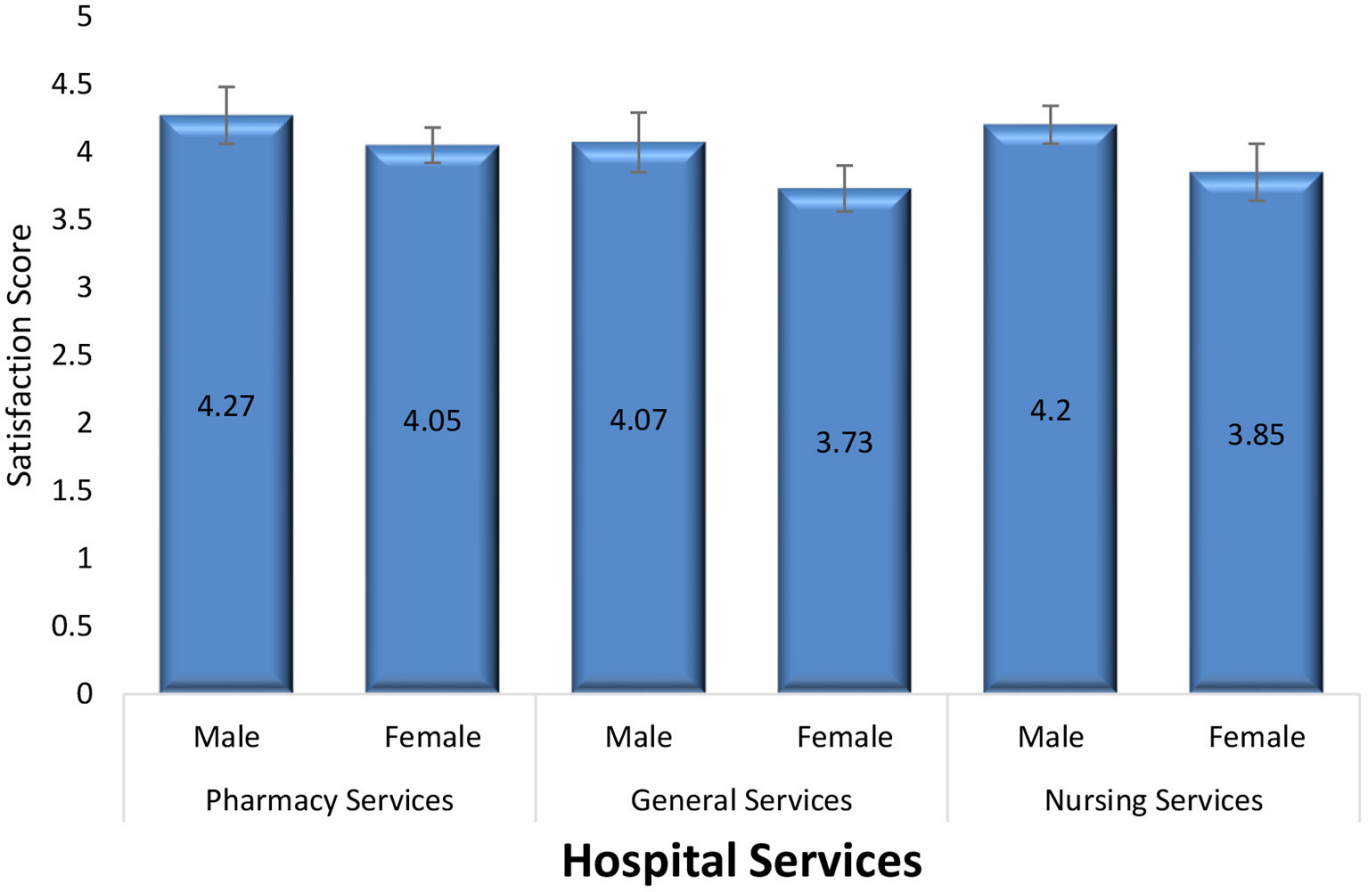
Comparison of satisfaction of pharmacy services with other hospital services.

As exhibited by Figure 1, satisfaction of patient with pharmacy services were more than nursing and general hospital services. However, the difference between these three services were not statistically significant.

### Patient satisfaction survey using PSQ-18

All 18 items of evaluation in PSQ-18 scale were divided into seven categories as explained above in the methods section. The interpersonal manner was given maximum score and while the time to get the appointment of physician was rated least. The average of rating was 3.38 and 3.4 given by male and female participants, respectively.

**Figure 2:**
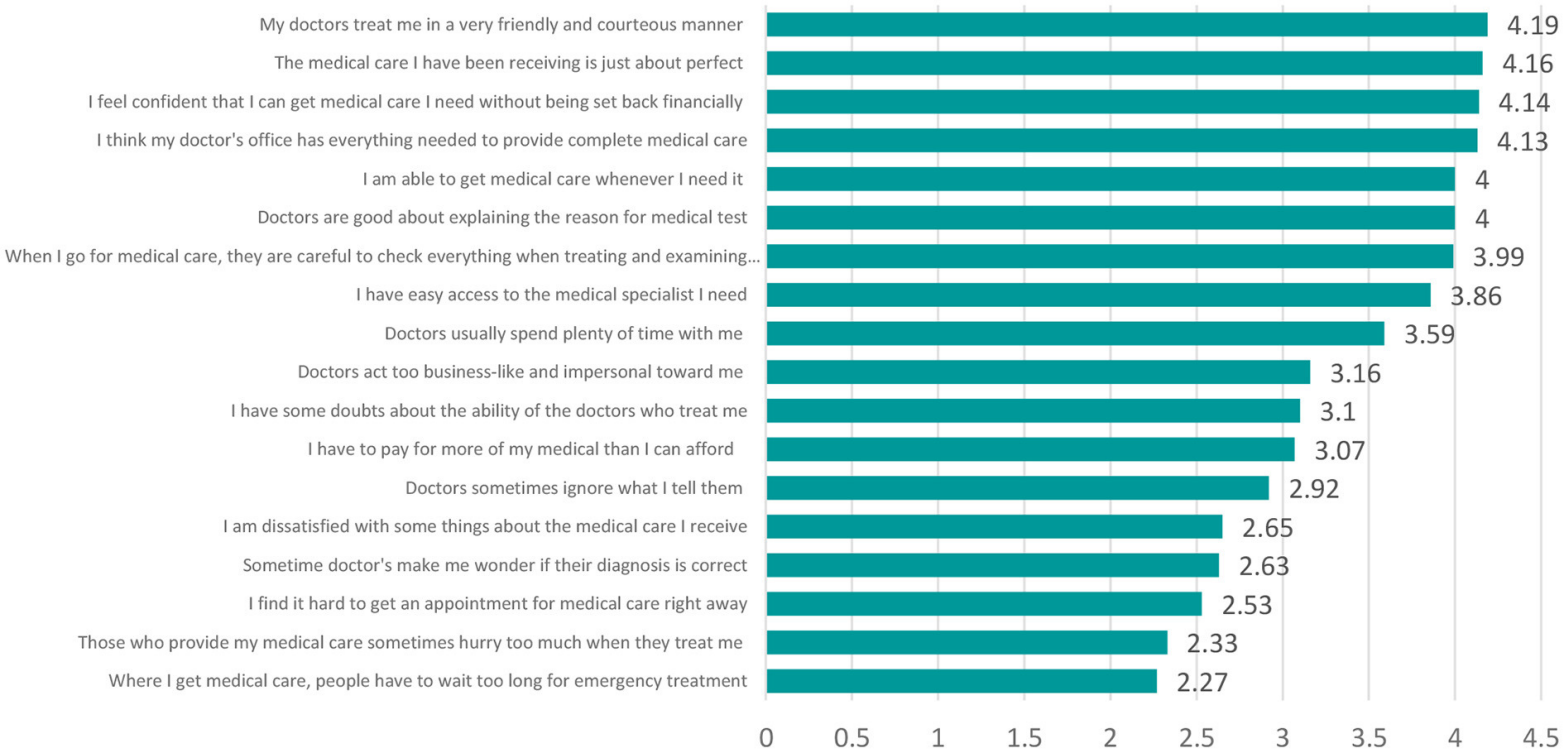
Comparison of scores given by participants of the study on PSQ-18 items.

As shown in Figure 2, highest score was obtained for item where participants were asked about the friendly nature of the physician, more than 80% of the score was accorded for this statement, whereas lowest rating was given to the time patient need to spend for getting the appointment of the physician. The average rating of 18 items were 3.37 which is unfortunately lower than the minimum expected rating of 70%.

The patient satisfaction questionnaire (PSQ-18) has a mean satisfaction score of 3.37. So, the satisfaction score was divided into two categories: satisfaction (≥3.37), and dissatisfaction (< 3.37). The logistic regression analysis of the variables influencing the ratings on the PSQ-18 scale is described in Table 7 (outcome variable). When compared to patients who were not employed, the odds of being satisfied were 2.4 times higher for those who were employed, but the odds of being satisfied were inversely correlated for students. Participants with higher levels of education expressed 2.1 times more satisfaction with the hospital's services than participants with lower levels of education. Participants between the ages of 18 and 35 years demonstrated 1.51 times the odds of satisfaction compared to participants over the age of 50. Patients between the ages of 36 and 50 exhibited a poor correlation with satisfaction. In addition, as compared to their female counterparts, male participants expressed 1.41 times higher levels of satisfaction with the hospital's overall services.

**Table 7:** Bivariate and multivariable logistic regression analysis of factors influencing patients' satisfaction on PSQ-18 items (258).

**Variables**	**Frequency (%)**		**AOR (95% CI)**	***P* value**
	**Satisfied** **[158 (61.2)]**	**Dissatisfied** **[100 (38.75)]**		
**Gender**	
Male	102 (64.5)	68 (68)	1.41 (1.02, 2.21)	0.067
Female	56 (35.4)	32 (32)	1.0	
**Age**	
18–35	106 (67)	50 (50)	1.51 (1.03,2.89)	0.045*
36–50	42 (26.5)	33 (33)	0.78 (0.54,1.23)	0.96
>50	10 (6.3)	17 (17)	1.0	
**Educational level**	
Higher	129 (81.7)	61 (61)	2.1 (1.8, 3.2)	0.02*
Secondary	29 (18.3)	39 (39)	1.0	
**Marital status**	
Married	77 (48.7)	55 (55)	0.81 (0.7, 1.8)	0.31
Single	81 (51.2)	45 (45)	1	
**Employment status**	
Employed	113 (71.5)	26 (26)	2.4 (1.7,3.2)	0.015*
Student	31 (19.6)	40 (40)	0.56 (0.32,1.2)	0.23
Non-employed	14 (8.8)	34 (34)	1	

1, showed reference used. *Showed a P < 0.05 in the multivariate analysis and statistically significant, CI (Confidence interval).

## Discussion

This study was done to examine the level of satisfaction with the healthcare services offered at the tertiary care hospital in Riyadh, Saudi Arabia, among mentally ill patients. Of the study subjects, 70, 76.3, 83.3, and 61.2% were satisfied with general hospital, nursing, pharmacy, and overall medical services, respectively. The outcome of the study indicates that patients are generally satisfied with the facilities provided in mental healthcare, especially nursing care and pharmacy services.

In comparison to studies conducted in psychiatric settings elsewhere, such as 81.3% in Ethiopia (23) and 72% in Pakistan (29), our study found that general medical services were less satisfied (61.2%). This discrepancy may be brought on by using different tools (PSQ-III in Pakistan) for measuring satisfaction; variations in patient characteristics; various data administration techniques; diverse research designs; variability in mental health literacy; mental health services; and sociocultural differences. Contrarily, our study's satisfaction rating was higher than those reported from Egypt (31.2%) in psychiatric departments (21) and 44.8% in Los Angeles, California, in primary care clinics for anxiety disorders (30). The difference in the types of mental disorders (anxiety disorders in Los Angeles) and the assessment equipment and settings (primary health setting in the Los Angeles study), study design, and sociocultural factors may be the causes of the potential difference in those studies. Additionally, our study is comparable to research from Ethiopia (22) and Austria (31) with a respective satisfaction rate of 61.2% and 60%. In general, most patients were dissatisfied with the amount of time they spent with the doctor. They typically book a visit well–in advance and anticipate a longer conversation with the doctor, but this is not practical given the doctor's hectic schedule. The majority of PSQ18 survey items obtained average ratings altogether, but when measured against other domains, the financial and interpersonal domains performed better. The amount of time patients must wait for emergency care infuriates them. They have voiced concerns over the diagnoses made by doctors. The inability to schedule doctor appointments and the doctor's lack of interest in their issues also rankle with them. In earlier studies, it was shown that taking in information, listening, empathy toward the patient, emotional support, friendliness, explanation of medical therapy, and respect for the patient were all significant predictors of patient satisfaction (32). Furthermore, physicians' use of medical terms without clarifying their meaning has a detrimental effect on patient satisfaction (33). In terms of the right to respect, studies carried out in developing nations have shown that patients readily accept doctors' disrespect. It can be an instance of the “paternalistic approach,” a historical paradigm that believes that patients are inferior to doctors (33).

We explored the satisfaction rate of patients from a psychiatric clinic using the general hospital facilities that included reception, registration, and infrastructure. The study was conducted in a tertiary care hospital that is government-run and under the direct control of the Ministry of Health (MOH). The ministry of health is committed to providing the best service to the country's citizens and eligible residents, as evidenced by the high level of satisfaction stated by the study's participants, particularly about the infrastructure and overall atmosphere provided at the hospitals. Most of the participants in this survey were extremely pleased with the overall hospital amenities, particularly the cleanliness, tranquility, and availability of patient privacy. The participants' ratings of general hospital facilities in this study are slightly lower than those reported earlier from Southern Saudi Arabia (27) where the satisfaction level was 79.6%, while we found only 70% of patients satisfied with hospital facilities. This discrepancy could be due to the differences in the hospital settings (they used both hospital and primary health care centers) and the study participants.

All three of the nursing care-related items were significantly rated as outstanding by the participants. This shows that the nursing care received by the patients at these facilities is of the highest caliber. The results showed that 76.3% of participants were pleased with the nurses' kindness and respect. Most of them also commend the nurse's capacity for attentiveness. This finding is consistent with the findings of Joolaee et al. (34). The most important elements for patient satisfaction, according to the researchers, were simple availability to nurses and the nurses' attitude. The results of the current study on nurse satisfaction could be linked to ongoing education initiatives at government-run hospitals that are conducted to improve nurses' communication abilities. In addition to providing information and fostering communication between patients, families, and other members of the care team, nurses are uniquely positioned to comprehend the needs and preferences of patients and their families (35) whose expectations matter more than actual needs when assessing patient satisfaction. The outcome of this study emphasizes the importance of the ability to communicate. Patients were satisfied with the communication skills of the nursing fraternity at the psychiatric clinic of a tertiary care hospital.

The results showed that more than 83.3% of the participants were happy with the pharmacists' help in resolving drug-related problems. This satisfaction rate was high in contrast to earlier studies from other sources. Yang and colleagues discovered that 34% of South Korean patients in their study were satisfied (36). Geffen and colleagues (37) conducted a study in the Netherlands and discovered that 42% of patients were satisfied with basic pharmacy services. The gap may be caused by the expense of medical care; in other nations, patients might be obliged to pay for their prescriptions, whereas treatment is free at the hospital where this survey was done.

Regarding the elements that are linked to patients' satisfaction with healthcare services, we found that educated, employed, those between the ages of 18 and 35, and males had higher levels of satisfaction than less educated, single, participants over the age of 50, and females. In line with an earlier study (9), we observed that university-educated participants in our study samples were 2.23, 2.63, 2.82, and 2.1 times more satisfied with hospital facilities, nursing, pharmacy, and medical services. This might be because individuals with higher levels of education are more likely to be aware of the hospital's services than participants with lower levels of education. Another intriguing finding of this study was the high level of satisfaction among individuals who were employed. When compared to study subjects who were not working, we reported that employed participants were 2.5, 2.65, 2.82, and 2.4 times more satisfied with the general services provided by hospitals, nursing, pharmacies, and medical services, respectively. Our results are consistent with those of Aloh et al. (38) who found a significant relationship between employment status and patients' overall satisfaction with the standard of care provided at tertiary care institutions in Southeast Nigeria. Also, we found that participants under the age of 50 were more satisfied with the hospital's services than individuals beyond the age of 50. This may be the case because patient satisfaction rates may diminish with age due to health status deterioration (39). Additionally, we discovered that men were more likely than women to be satisfied with the hospital's amenities. Men and women may use services in different ways, have diverse perceptions, and have distinct needs and expectations from the healthcare services available. These disparities may be reflected in the differences between men and women's levels of satisfaction. The result of this investigation is consistent with the past report (22).

The current study has some limitations. It is challenging to determine the timing of the association between the patient group and the contributing factors because the study was originally cross-sectional. Second, because the study was carried out in a hospital, the social desirability bias might have had an impact. Additionally, there is a risk that there will be other confounding factors that affect the study's findings.

## Conclusion

The findings of this study revealed overall satisfaction with the hospital facilities, nursing care, pharmacy services, and medical care provided at the tertiary care hospital in Riyadh, Saudi Arabia. The general hospital services were positively correlated with factors including greater education, employment, younger age group, and male gender. The satisfaction with the pharmacy services were rated higher than the nursing care and hospital facilities. Expectations are not met in terms of customer satisfaction with the medical professionals' services. To ensure that patients, especially those with psychiatric illnesses, receive the best care possible, it is imperative to increase awareness of the value of improving patient satisfaction among all members of the healthcare team.

## Data availability statement

The original contributions presented in the study are included in the article/supplementary material, further inquiries can be directed to the corresponding author.

## Ethics statement

The studies involving human participants were reviewed and approved by Research Committee, AlMaarefa University. The patients/participants provided their written informed consent to participate in this study.

## Author contributions

Under the supervision of SA, MA, and WA carried out the research methodology. OA, MS, and ASA were responsible for formal analysis of the work while WA, ASA, MA, and AA participated in writing original draft of the manuscript. MA administered the project. SA was instrumental in review and editing of the manuscript. All authors contributed to the article and approved the submitted version.

## Funding

The authors extend their appreciation to the Deputyship of Research and Innovation, Ministry of Education in Saudi Arabia for funding this research work through the project number (1-442-50).

## Conflict of interest

The authors declare that the research was conducted in the absence of any commercial or financial relationships that could be construed as a potential conflict of interest.

## Publisher's note

All claims expressed in this article are solely those of the authors and do not necessarily represent those of their affiliated organizations, or those of the publisher, the editors and the reviewers. Any product that may be evaluated in this article, or claim that may be made by its manufacturer, is not guaranteed or endorsed by the publisher.
